# Sub-phenotyping of juvenile dermatomyositis: can it assist clinical decisions?

**DOI:** 10.1186/1546-0096-12-S1-O18

**Published:** 2014-09-17

**Authors:** Shireena A Yasin, Katie Arnold, Erdal Sag, Sarah Tansley, Elena Moraitis, Thomas S Jacques, Janice L Holton, Catherine Owens, Neil McHugh, Clarissa Pilkington, Lucy R Wedderburn

**Affiliations:** 1 Infection Inflammation and Rheumatology Section, Institute of Child Health, UCL, London, UK; 2 Rheumatology, Royal National Hospital for Rheumatic Diseases, Bath, UK; 3 Birth Defects Research Centre, Institute of Child Health, UCL, UK; 4 Molecular Neuroscience, MRC centre for neuromuscular diseases, Institute of Neurology, UCL, UK; 5 Cardiothoracic Radiology, Great Ormond Street Hospital, London, UK; 6 Rheumatology, Great Ormond Street Hospital, London, UK

## Introduction

Juvenile Dermatomyositis (JDM) is a rare serious disease (affecting 2-3 million children/year) presenting with rash and proximal muscle weakness. Serious complications can include calcinosis, GI ulceration, interstitial lung disease (ILD) and even death. It is becoming clear that JDM is a heterogeneous condition. Dividing JDM into sub-phenotypes would allow better prediction of disease severity and more targeted treatments. We have identified novel auto-antibodies in subtypes of JDM that may correlate with specific phenotypes.

## Objectives

To define clinical & pathological phenotypes of patients with JDM who have antibodies to Melanoma Differentiation-Associated protein 5 (MDA-5).

## Methods

### Patients

Patients were included from the Juvenile Dermatomyositis Cohort and Biomarker Study, a multi-centre study including 13 centres from across the UK. The study collects longitudinal clinical and serological data from patients with idiopathic inflammatory myopathies (IIM) of which 85% are diagnosed with JDM or JDM overlap features (currently n=446 patients). Clinical data collected included presence of clinical features, treatment, physicians global assessment and muscle strength assessments including the Childhood Muscle Assessment Score (CMAS).

### Autoantibodies

Plasma or serum, available for 285 patients, were screened for the presence of autoantibodies by immunoprecipitation and confirmed by ELISA using recombinant MDA-5 protein.

### Muscle biopsies

Muscle biopsies were stained and scored using the JDM Muscle biopsy score tool as described (1,2) .The validated muscle biopsy score tool measures severity of muscle pathology across 4 domains and with a separate visual assessment score (0-10).

## Results

Autoantibody screening identified the presence of MDA-5 antibodies in 7.4% of patients (21/285 cases). MDA-5 positive patients had significantly increased incidence of ulceration (p=0.03), arthritis (p<0.01) and lung disease, yet had less severe muscle involvement, measured by CMAS score (p=0.03), than MDA-5 negative patients. In addition, median muscle biopsy scores for the MDA-5+ve patients were significantly lower than the MDA-5-ve patients (p<0.005) suggesting a less severe muscle pathology.

## Conclusion

JDM is a heterogeneous condition with sub-phenotypes defined by autoantibody status, clinical features and muscle pathology. Identification and classification of sub-phenotypes could be used to predict disease course and severity. In the future, JDM specific autoantibodies could be used as biomarkers allowing for stratified approaches to treatment.

## Disclosure of interest

None declared.

